# Epidemiology and costs of cervical cancer screening and cervical dysplasia in Italy

**DOI:** 10.1186/1471-2458-9-71

**Published:** 2009-02-25

**Authors:** Paolo Giorgi Rossi, Alessandro Ricciardi, Catherine Cohet, Fabio Palazzo, Giacomo Furnari, Sabrina Valle, Nathalie Largeron, Antonio Federici

**Affiliations:** 1 Agency for Public Health, Lazio Region, Via di S. Constanza 53, 00198 Rome, Italy; 2 Sanofi Pasteur MSD, 8 rue Jonas Salk, 69007 Lyon, France; 3 National Research Council, Institute for Research on the Population and Social Policies, Via Nizza 128, 00198 Rome, Italy; 4 Centre for Disease Control, Ministry of Health, Via della Civiltà Romana 7, 00144 Rome, Italy

## Abstract

**Background:**

We estimated the number of women undergoing cervical cancer screening annually in Italy, the rates of cervical abnormalities detected, and the costs of screening and management of abnormalities.

**Methods:**

The annual number of screened women was estimated from National Health Interview data. Data from the Italian Group for Cervical Cancer Screening were used to estimate the number of positive, negative and unsatisfactory Pap smears. The incidence of CIN (cervical intra-epithelial neoplasia) was estimated from the Emilia Romagna Cancer Registry. Patterns of follow-up and treatment costs were estimated using a typical disease management approach based on national guidelines and data from the Italian Group for Cervical Cancer Screening. Treatment unit costs were obtained from Italian National Health Service and Hospital Information System of the Lazio Region.

**Results:**

An estimated 6.4 million women aged 25–69 years undergo screening annually in Italy (1.2 million and 5.2 million through organized and opportunistic screening programs, respectively). Approximately 2.4% of tests have positive findings. There are approximately 21,000 cases of CIN1 and 7,000–17,000 cases of CIN2/3. Estimated costs to the healthcare service amount to €158.5 million for screening and €22.9 million for the management of cervical abnormalities.

**Conclusion:**

Although some cervical abnormalities might have been underestimated, the total annual cost of cervical cancer prevention in Italy is approximately €181.5 million, of which 87% is attributable to screening.

## Background

Cervical cancer is the second most common cancer in women worldwide and accounts for almost 10% of all cancer-related deaths. In Italy, it is estimated that each year 3,418 new cases and 1,186 deaths occur [1]. Infection with Human Papillomavirus (HPV) has been established as the necessary cause of cervical cancer [2,3]. As this cancer can be diagnosed at an early stage, screening programs using the Papanicolaou (Pap) smear test have reduced significantly the number of invasive cases, through the diagnosis and treatment of precancerous lesions (cervical intraepithelial neoplasia, [CIN]) [4,5].

The Italian Ministry of Health establishes health-related objectives at national level, while implementation is the responsibility of regional governments. Some regions started implementing cervical cancer screening programs in the 1970s, while nationwide organized programs, rolled out on a regional basis, were recommended in 1996, based on the European Commission Guidelines on Quality Assurance in Cervical Cancer Screening [6]. By 1999, however, only 34% of Italian women aged 25–64 years were included [7]. In 2004, the Italian Government ordered that each region should plan and implement cervical cancer screening programs [8], to be provided by Local Health Authorities. Such programs are still not fully implemented across the country, although opportunistic screening has increased. Two preventive cervical cancer vaccines (Gardasil^® ^and Cervarix^®^) are now registered in Italy. In March 2008, a mass vaccination campaign targeted at eleven-year-old girls was initiated, with some regions vaccinating a second cohort of girls (14 or 15 year-olds) and one region introducing a multi-cohort vaccination strategy, focused on four cohorts of girls aged 11, 14, 17 and 24 years.

In the absence of national data on the epidemiology of cervical cancer screening in Italy, the present study was undertaken to provide an estimation of the number of women undergoing screening annually, the annual incidence of cervical dysplasia, and direct costs associated with the management of women diagnosed with cervical dysplasia.

## Methods

### Estimation of the annual number of Pap smears in Italy

While organized screening programs publish the results of their activity periodically, information on opportunistic screening, i.e. Pap tests performed by public and private providers but not included in a screening program, is scarce. An estimation of the total number of women screened annually was obtained from a survey performed by the National Institute of Statistics (ISTAT) [9], which provides data on Pap test use and screening intervals by age and geographic area. The number of women screened by screening interval was calculated by multiplying the coverage (percentage) by the number of women aged 25–69 years resident in Italy in 2005. To check the consistency of our estimate, we used two recently published studies to calculate the interval of screening (not shown) [10,11]. An alternative method, integrating data from the Italian Group for Cervical Cancer Screening (GISCi) for compliers and the ISTAT estimates for non-compliers, was used to obtain a lower bound in sensitivity analysis; this second hypothesis is based on the assumption that no women had a Pap test (neither from organized nor from opportunistic settings) in the three-year interval.

### Estimation of the annual number of abnormal Pap smears

Since 1997, the GISCi has been conducting surveys to assess screening programs, which until 2006 collected data on Pap tests and their results using the Bethesda System. The proportion of unsatisfactory samples and positive tests (including atypical squamous cells of undetermined significance [ASCUS], low-grade squamous intraepithelial lesions [LSIL], high-grade squamous intraepithelial lesions [HSIL] and suspected carcinoma) was obtained from the 2004 GISCi survey [11], and applied to the estimated annual number of Pap smears. To calculate the number of women referred for a repeat Pap smear, the proportion of unsatisfactory smears (6.1%) was applied to the total annual number of Pap smears, and then multiplied by the compliance rate for repeated cytology (62.2%), as observed in the GISCi survey [11].

### Management of abnormal cytology and treatment of pre-invasive lesions

To establish the typical management of abnormal cytology and the management of cervical dysplasia, Italian and international guidelines for cervical cancer screening were reviewed. Published guidelines and protocols outlining details of patient management were identified through Medline Entrez-PubMed using the following search criteria: 'uterine cervical neoplasms', 'uterine cervical dysplasia', 'cervical intraepithelial neoplasia' AND 'screening'; the search was limited to 'practice guidelines'. In addition, a search of Embase and the Cochrane database was conducted using the search terms 'uterine cervix cancer' and 'uterine carcinoma in situ'.

The initial Medline search, concluded on 30 July 2006, gave 77 hits; a review of corresponding abstracts excluded 57 irrelevant, non-English or non-Italian articles. The Embase search did not add any new item. The reference sections of each identified article were checked for further guidelines (one additional item found). A screening of governmental agencies and Italian scientific societies identified 12 additional items. Agencies include the Italian National Health Agency (AgeNaS) and regional websites of Emilia Romagna, Piemonte, Veneto, Toscana, Lazio, Basilicata. The web database of the following international and national agencies was reviewed: IARC (International Agency for Research on Cancer), NHS (National Health Service) Cervical Screening Programme and NICE (National Institute for Clinical Excellence) UK, ANAES (Agence Nationale d'Accréditation et d'Évaluation en Santé) France, National Cervical Screening Programme New Zealand. The following Italian scientific societies were identified: GISCi (Italian Group for Cervical Cancer Screening), ONS (National Centre for Screening Monitoring), AIE (Italian Association of Epidemiology), SICi (Italian Society of Cytology), SICPCV (Italian Society of Cervical Pathology and Colposcopy) and SIAPEC (Italian Society of Pathology and Cytodiagnostics).

### Estimation of the annual number of CIN1 and CIN2/3 diagnoses

In order to estimate the number of CIN1 lesions diagnosed each year, the CIN1 detection rate reported in the GISCi survey [11] was applied to the total number of Pap smears performed annually, although only 40 centres out of 95 organized cervical cancer screening programs participating in the GISCi survey registered data on CIN1 [12].

Two methods were used to estimate the number of CIN2/3 lesions. Method 1 was based on data from the Emilia Romagna Cancer Registry [13], the only Italian cancer registry providing reliable data on pre-invasive lesions. The detection rate was calculated by dividing the number of lesions by the estimated number of women screened in the same area and time period. This rate was then applied to the estimated total number of Pap smears performed annually in Italy. Method 2 applied the detection rate of CIN2/3 from the GISCi survey [11] to the total annual number of Pap smears. Currently, the number of CIN2, CIN3 and invasive carcinoma are reported in 75 out of 95 organized cervical cancer screening programs in Italy. The relative proportions of such lesions that were attributable to CIN2 and CIN3 were determined according to the results of the GISCi survey of second level screening for cervical cancer [12]. Both sources, the survey and the cancer registry, collect histological results according to the Richart classification for CIN.

### Costs associated with cervical screening and treatment of cervical dysplasia

To calculate the total annual cost of cervical cancer screening from the healthcare perspective, unit costs for the relevant procedures were applied to the estimated numbers for total Pap tests and for women with each type of abnormal cervical cytology, based on the National Tariff Formularies [14] (Table 1). For women who had Pap tests performed within a screening program, there was an additional cost pertaining to each test invitation [[15]; ASP Lazio, unpublished data]. A cost for gynaecologic examination was added to the cost of Pap tests performed outside the screening program. Disruptive treatments for CIN (e.g. laser vaporization, cryotherapy, diathermocoagulation) were considered outpatient procedures.

**Table 1:** Unit costs for invitation, diagnostic procedures and treatments.

**Procedure**	**Formulary code** **(Lazio region)**	**Tariff (€)**
Invitation for screening	(not included in formularies)	3.33
		
Diagnostic		
Pap test (conventional smear)	91.38.5	11.16
HPV test	91.37.01	81.60
Colposcopy	70.21	10.74
Endocervical biopsy	67.11	24.79
Histological evaluation of vaginal biopsy	91.45.5	14.10
Gynecologic examination	89.26	13.63
Treatments		
Disruptive		
Laser vaporization	67.39	None
Cryotherapy	67.33	37.18
Excisional		
Leep, cold blade and surgical conisation	DRG code 630	
Day hospital		913.85
Full hospitalization		1,516.84

Leep: loop electrical excision procedure

It was assumed that excisional treatments for CIN (radiofrequency excision, cold-knife excision and laser conisation) were performed in hospital, either as day-hospital or inpatient procedures, and costs for these procedures were obtained from the Lazio Hospital Discharge Registry (Table 1). All costs are reported in 2005 euros.

### Sensitivity analysis

A sensitivity analysis was performed for five parameters:

1. For the total number of Pap tests, we used as lower bound of the estimate the result of a mixed method that includes the GISCi survey data and excludes overlaps between organised and opportunistic screening in the three-year period.

2. For the number of CIN2/3, we used the extrapolation from the GISCi survey as the higher bound of the estimate.

3. We varied the rate of referral to colposcopy from the value observed for the 25^th ^centile of the programmes distribution of the GISCi survey to the 75^th ^centile of this distribution.

4. We report the result of a hypothetical massive introduction of HPV testing for the triage of ASCUS, using the following assumptions: all ASCUS are referred to HPV test; the compliance to HPV test is 89% (as for colposcopy); the proportion of HPV positive among ASCUS is 24.2% [16]; the proportion of CIN1/2/3 consequent to ASCUS diagnosis among the total CIN1/2/3 in ASCUS + LSIL is 41% [16]; the biopsy rate among ASCUS HPV+ is arbitrarily set to 90%; the HPV test cost is 81.60€ as national tariff and arbitrarily 15€ in the prevision of a strong price reduction.

5. Since the tariff of colposcopy is one of the lowest in industrialised countries, we report the total costs under the assumption that in opportunistic screening, colposcopy is always associated to a gynaecological visit.

6. We report the total costs under the assumption of an inverted proportion of CIN1 treated (i.e. 63% instead of 37%).

7. We report the total costs under the extreme hypothesis of all conisations performed in outpatient settings. This does not apply to the conisations for recurrence.

## Results

### Estimation of the annual number of Pap smears in Italy

Our calculations suggest that a total of 6,423,924 women aged 25–69 years undergo screening on an annual basis in Italy (Table 2). According to ISTAT, there are approximately 16.5 million Italian women aged 25–64 years and therefore eligible for organized cervical screening. Given the recommended 3-year screening interval, approximately 5.5 million women (one-third of the target population) should be invited for cervical screening each year. However, screening programs only contact approximately 2.8 million women each year and 1.2 million of these women are actually screened [11]. These latter figures correspond to 27.5% and 11.8%, respectively, of the female target population living in areas having active screening programs. Although the number of projected screened women includes some women beyond the recommended age for screening, it can be estimated that approximately 5.2 million women in Italy undergo opportunistic screening.

**Table 2:** Estimated annual number of Pap smears in women aged 25–69 years, Italy 2005.

	**Number**	**%**
Total number of women	6,423,924	100.00
Satisfactory Pap smears	6,034,550	93.9
Unsatisfactory Pap smears	389,376	6.1
Repeat Pap smears	242,194	--
Total number of Pap smears	6,666,118	--

From a total of approximately 6.4 million women who undergo screening, it is estimated that about 390,000 have unsatisfactory results and, as a consequence, approximately 240,000 have a repeated test (Table 2). Overall, approximately 6 million women have a cytological result that permits assessment of their risk of cervical cancer.

### Estimation of the annual number of cytological abnormalities

According to the GISCi survey 2004, 2.4% of Pap tests were positive [11]. By applying this proportion to the estimated number of satisfactory tests, we estimate that 153,393 women have a positive result (Table 3). The most frequent finding is ASCUS, followed by LSIL.

**Table 3:** Estimated cytology results for women aged 25–69 with a satisfactory Pap smear, Italy 2005.

**Cytology finding**	**n**	**% Positive**	**% Diagnosis**
Total number of satisfactory cytological diagnoses	6,276,744	100.0	--
Negative	6,123,349	97.6	--
Positive	153,393	2.4	100.0
ASCUS	68,161	1.09	44.4
LSIL	44,539	0.71	29.0
HSIL	11,976	0.19	7.8
Carcinoma	678	0.01	0.4
Other^1^	28,039	0.45	18.3

^1^Includes women referred for colposcopy after two unsatisfactory Pap smears

### Management of women with abnormal cytology

Twenty nine guidelines about screening and management of abnormal Pap tests were identified and reviewed. All current Italian guidelines recommend the TBS classification for cytology results (version 1991 followed by 2001). The management of LSIL, HSIL and suspected carcinoma is clearly defined: colposcopy and, if positive, biopsy. Conversely, ASCUS has three acceptable management options: colposcopy, repeat cytology at 6 months or HPV triage. On the basis of the GISCi data [11] and expert opinion, only direct referral to colposcopy was considered for the management of ASCUS. For HSIL, it was assumed that 90% of cases would undergo biopsy, but overall 49.6% of abnormal cases referred for colposcopy also underwent biopsy, as reported by GISCi [11]. We estimated that, of the 153,393 women with abnormal cytology, 134,054 would undergo colposcopy and 66,450 would have a biopsy.

There is no consensus guideline on the duration and frequency of follow-up of negative findings by colposcopy and biopsy. It was assumed that the typical follow-up for women with negative findings by colposcopy after LSIL or less severe cytological results was a Pap test after one year. The typical follow-up for negative colposcopy findings after HSIL or more severe cytological results was assumed to be two colposcopies with a repeated Pap test.

### Cost of cervical screening and management of abnormal Pap tests

The total cost of cervical screening in Italy was estimated to be €158.5 million annually (Table 4). Most of these costs were associated with opportunistic screening. The costs of additional tests in women with an abnormal Pap smear were estimated to be approximately €4.0 million in the first year and €5.2 million including the costs of follow-up (Table 5). Most of these costs were attributable to women with ASCUS/LSIL. However, the unit cost per patient was higher for HSIL/carcinoma than for ASCUS/LSIL (Table 5).

**Table 4:** Estimated annual costs of detection and management of cervical dysplasia (including follow-up), Italy 2005.

	**Annual cost (€)**
Total screening costs	158,541,733
Screening program	
Cost of invitations	9,293,041
Cost of Pap smears	13,104,094
Opportunistic screening	130,140,608
Repeated Pap smears	6,003,989

**Table 5:** Estimated total and unit costs for diagnosis of abnormal Pap smears and treatment of CIN, Italy 2005.

	**First year**		**Total cost** **(First year and follow-up)**	
	**Total cost (€)**	**Unit cost (€)**	**Total cost (€)**	**Unit cost (€)**
Diagnosis	3,986,007	20.1	5,179,506	33.8
ASCUS/LSIL	2,780,336	24.7	3,595,139	31.9
HSIL/carcinoma	499,034	41.7	602,402	50.3
Others	706,637	24.6	981,965	34.2
CIN treatment	16,381,037	579.1	17,779,760	628.5
CIN1^1^	4,819,842	226.2	5,580,319	261.9
CIN2^2^	4,336,532	1347.7	4,630,430	1439.1
CIN3^2^	7,224,664	1919.4	7,569,011	2010.9

Total	20,367,044		22,959,267	

^1^Estimate based upon data from the GISCi 2004 survey [12]; ^2^Estimate based upon data from the Emilia Romagna Cancer Registry [13].

### Estimation of the annual number of CIN1 and CIN2/3 diagnosed in Italy

It was estimated that 21,308 CIN1 lesions are diagnosed each year in Italian women. Estimates based on data from the Emilia Romagna Cancer Registry (Method 1) suggested that 6,982 CIN2/3 lesions are diagnosed annually: 3,218 cases of CIN2; 3,518 cases of CIN3; and 245 cases of adenocarcinoma *in situ *(AIS). These data excluded women aged less than 24 years, for whom there is no estimate. The GISCi survey (Method 2) provided a substantially higher estimate for the total incidence of CIN2/3 of 16,571 cases (7,638 CIN2; 8,350 CIN3; 583 AIS) and 738 invasive cancers, resulting in a detection rate of 2.7/1000. The estimated incidence of AIS and invasive adenocarcinoma is lower than for squamous cell lesions. It was estimated that there would be 274 cases with recurrent CIN2 and 333 cases with recurrent CIN3 during a 2-year follow-up period.

### Management of cervical dysplasia (CIN)

Sixteen guidelines about treatment of pre-invasive lesions were identified and reviewed. All current Italian guidelines recommend the Richart (CIN) classification for histology. A few Italian guidelines specifically refer to the treatment of CIN lesions. These include the Italian Society of Cervical Pathology and Colposcopy, the Italian Society for Gynaecology and Obstetrics (SIGO), the Society for Gynaecology and Obstetrics of Lombardy (SLOG) and those of the Emilia Romagna and Tuscany Regions. A review of these guidelines revealed three points where management practices may vary. First, it is recommended that CIN1 lesions should be followed up and treated only if persistent, or alternatively when the colposcopy is incomplete or the lesion is not completely visible. However, the GISCi survey found that the proportion of treated CIN1 lesions varies extremely between different centres [12].

Consequently, the overall percentage of treated CIN1 lesions from the GISCi survey (37%) was used for all analyses. Secondly, guidelines recommend excision as the preferred treatment option for all grades of CIN, although the use of disruptive surgery is also allowed in some cases. Our estimates were based on the percentage of excisions and disruptive treatments observed in the GISCi survey for each CIN grade [12]. For CIN1, 63.0% of cases had no immediate treatment (follow-up cytology and colposcopy at 6 months), 20.3% had disruptive treatment (29% laser vaporization and 71% diathermocoagulation) and 16.7% had excisional treatments. For CIN2/3, 3.7% and 96.3% of cases had disruptive and excisional treatment, respectively. The third point of discrepancy between guidelines is the duration and intensity of follow-up for CIN2/3 after treatment. It was assumed that all women would have had a repeat Pap test and colposcopy every 6 months for 2 years, as recommended in the guidelines from the Emilia Romagna Region. The estimated proportion of cases with recurrent CIN after treatment for CIN2/3 was based on findings reported in the study by Cecchini [17]. Any controversial points were discussed with an expert panel.

### Costs of cervical dysplasia treatment

Using the lower estimate for the incidence rate of CIN2 and CIN 3, the total annual cost of treating CIN was €16.4 million, excluding any follow-up costs, and €17.8 million when follow-up costs were included. Treatment of CIN3 accounted for almost half of all costs (Table 5). The overall unit costs per patient were estimated to be €579 and €628, excluding and including costs of follow-up, respectively. The unit cost increased with disease severity.

If the higher estimates for incidence of CIN2 and CIN3 are used, the estimated costs of treatment (including follow-up) increase to €11.8 million and €19.3 million, respectively, and the overall cost of managing CIN could be as high as €36.7 million.

The combined cost of diagnosis and treatment of cervical lesions is €22.9 million, if the costs of follow-up are included (Table 5). When the costs of screening are included, the total annual cost of the cervical screening program amounts to €181.5 and €200.5 million, respectively, when the lower and the higher CIN2/3 incidence rates are considered.

### Sensitivity analysis

The most influential parameters are the number of CIN2/3, which increases the total cost by 6.4%, and the setting for conisation, that can reduce the total costs up to 11.1% (Table 6).

**Table 6:** Univariate sensitivity analysis of screening, abnormal findings management and treatment costs (€), Italy 2006.

	**Screening **	**Abnormal findings and treatment **	**Total costs of cervical cancer prevention**
**Baseline values for all the parameters**	**158,288,703**	**22,959,267**	**181,247,970**
No. of screened women yearly			
baseline: extrapolation from ISTAT (N = 6,423,924)			
lower case: similar, but excluding overlap between opportunistic and organised screening (N = 6,270,130)	154,332,393	22,825,230	177,157,624
No. of CIN2/3 found			
baseline: estimate from Emilia Romagna cancer registry (N = 6,982)			
higher case: extrapolation from GISCi survey (N = 16,571)	158,288,703	34,532,811	192,821,515
Referral rate			
baseline: extrapolation from GISCi survey (2.5%)			
lower case: 25th centile of the GISCi survey distribution (1.5%)	158,288,703	20,920,849	179,209,552
higher case: 75th centile of the GISCi survey distribution (3%)	158,288,703	24,235,135	182,523,838
ASCUS management			
baseline: no triage with HPV test			
triage with HPV test (cost 81.60€ according to national tariff)	158,288,703	26,101,216	184,389,919
triage with HPV test (cost 15.00€)	158,288,703	22,197,251	180,485,954
Colposcopy setting			
baseline: no gynaecological visit			
upper case: gynaecological visit for colposcopies performed in opportunistic screening	158,288,703	25,199,706	183, 488,409
CIN1 treatment			
baseline: 37% of CIN1 treated			
upper case: 63% of CIN1 treated	158,288,703	26,231,936	184,510,639
Conisation setting			
baseline: inpatient (36% day hospital and 64% full hospitalization)			
lower case: outpatient	158,288,703	8,084,711	166,373,414

## Discussion

Our study suggests that costs associated with the detection and treatment of cervical dysplasia are substantial. Each year the Italian healthcare service is expected to spend €181.5 million (€200.5 million if the higher incidence of CIN2+ lesions is taken into account) to screen approximately 6.4 million women aged 25–69 years, to manage more than 150,000 women with abnormal Pap smears, and to treat an estimated 21,000 women with CIN1 lesions and 7,000–17,000 women with CIN2/3 lesions. Although the majority of these costs are directly related to cervical screening, only a small proportion is attributable to organized screening programs, with opportunistic testing accounting for the greatest share of costs. In contrast, our estimates suggest that only 11% of the total healthcare costs are associated with the management of CIN lesions; this percentage increases to 18% if the higher CIN2/3 incidence rate is applied.

Interestingly, although approximately 6.4 million women have a Pap test each year, which should cover the target population for organized screening, there are still 30% of women who had no Pap test in the last three years. Only 1.2 million women are screened within organised programs, but approximately 5.2 million are screened opportunistically. This suggests that some women are screened more frequently than the recommended 3-year interval, while others are screened despite being older than the recommended age, whereas some women may never be screened. This is consistent with analyses from the ISTAT data, showing that up to 50% of women in Southern Italy and the Italian islands have never been screened, compared with approximately 25% of women in Northern and Central Italy [18].

This study has several limitations. Firstly, extrapolation of GISCi data to the whole Italian female population may result in an overestimation of the number of women screened and in an underestimation of CIN management. Secondly, although the GISCi survey represents the only available data relating to screening programs from different Italian regions, this data collection system neither includes opportunistic screening carried out in the private setting, nor takes into account data from each Italian region. Furthermore, some Italian regions do not yet have formalized screening programs, and data included in the GISCi survey 2004 [11,12] are, therefore, collected exclusively in regions with well-established programs. Thirdly, the absence of any national data on opportunistic screening, which accounts for the majority of screening in Italy, also limits estimates of the epidemiology of cervical dysplasia. Lastly, we could not estimate the number of cases of CIN2/3+ for women younger than 24 years of age and older than 70 years. Although a small proportion of CIN2/3+ occurs in women under 24 years of age, such lesions are very likely to regress [19] and, according to guidelines, should not be diagnosed [8]. Conversely, very few women over 70 years of age have Pap smears. Our calculations assume that *in situ *lesions detected in women older than 69 years, as reported by the Emilia Romagna Cancer Registry, can be attributed to Pap smears performed in women aged 65–69 years.

The two methods used to estimate the annual number of CIN2/3 lesions produced markedly different values. Method 1, based on data from the Emilia Romagna Cancer Registry, suggested that there are about 7,000 cases of CIN2/3 diagnosed. However, data drawn from a single registry cannot have 100% sensitivity to accurately project the incidence of these lesions nationwide, especially as this data collection is relatively new and does not have well-established quality indicators, such as for invasive cancer. The value derived using Method 1 can, therefore, be used as a lower limit of the CIN2/3 range. Method 2, which used data from the GISCi survey, suggested that there are more than 17,000 cases of CIN2+ diagnosed each year. This number might overestimate the incidence rate, as the detection rate observed in organized screening programs using a 3-year interval is probably higher than the detection rate of opportunistic screening, often based on a 1-year interval. This estimate can, therefore, be used as the upper limit of the CIN2/3 range. In our sensitivity analysis, this parameter appeared to be one of the most influential, resulting in the highest cost estimate (+ 6.3%).

With reference to the annual expected number of CIN1, it should be noted that only 42.1% (40 out of 95) of centres participating in the GISCi survey systematically provide data. Although these lesions do not generally require treatment, it is commonly agreed in clinical practice, particularly in opportunistic screening, that deviations from best practice are frequent and over-treatment is often performed. Consequently, the impact of CIN1 treatment on costs might have been underestimated.

In the cost calculations, it was assumed that all women with ASCUS would be referred directly for colposcopy, although this is not entirely consistent with current guidelines or clinical practice. However, the introduction of HPV triage does not change substantially the total costs, as shown by the sensitivity analysis. Nevertheless, estimation of the costs of additional diagnostic procedures for LSIL and HSIL and for treatment of CIN was based on practices reported in the GISCi survey, so may be considered representative of current practice in Italy.

The shift of treatment procedures to a more appropriate setting of care, in particular conisation from inpatient to outpatient is currently the most suitable way to reduce total costs of disease. Nevertheless, a change in the setting of surgery from day hospital to outpatient has a strong impact if tariffs are used to measure costs, but this is an overestimation of the real impact on the real costs sustained by the Health Service.

Although it is not possible to make direct cost comparisons between different healthcare systems, it is interesting to note that the costs of cervical screening in Italy are comparable with those for England, where approximately 4 million women are screened each year at a cost of €157 million (approximately €200 million), including the cost of treating abnormalities [20]. However, the estimated cost per case associated with CIN1 treatment in the present study was lower in comparison with the mean cost reported in England and Wales (€419; approximately €532), whereas the estimated unit costs for CIN2 and CIN3 were considerably higher than the mean costs (€572 [approximately €726] and €606 [approximately €770], respectively) [21].

As the focus of this study was the economic burden of cervical dysplasia, our calculations did not take into account societal costs, such as work loss due to screening or for CIN treatment. We also did not capture the psychological burden of receiving a positive Pap smear result.

Until recently, regular screening was the only tool to prevent cervical cancer. The development of HPV vaccines has provided a new opportunity to reduce the incidence of cervical dysplasia [22-24] and studies have shown that a combination of both vaccination and screening is a cost-effective solution to reduce morbidity and mortality caused by cervical cancer [25-28].

## Conclusion

This is the first study to assess the epidemiology of cervical cancer screening and cervical dysplasia, as well as the associated healthcare costs, for Italy as a whole. Each year, the Italian healthcare service system makes a substantial investment of €158.5 million for the provision of screening services and €22.9 million for the follow-up and treatment of cervical abnormalities.

Higher adhesion to guidelines and protocols would produce substantial savings. Although the number of Pap tests performed each year would be sufficient to cover the whole target population, 30% of women are still not screened.

The economic burden of disease associated with cervical dysplasia identified in this study will assist health authorities with both the planning and allocation of funding for an effective cervical cancer prevention strategy.

## Abbreviations

ASCUS: atypical squamous cells of undetermined significance; CIN: cervical intraepithelial neoplasia; GISCi: Italian Group for Cervical Cancer Screening (Gruppo Italiano Screening del Cervicocarcinoma); HPV: human Papillomavirus; HSIL: high-grade squamous intraepithelial lesions; ISTAT: National Institute of Statistics (Istituto nazionale di statistica); LSIL: low-grade squamous intraepithelial lesions.

## Competing interests

The Agency for Public Health (ASP Lazio) received an unrestricted grant from Sanofi Pasteur MSD to conduct the present study. Results of this study were analysed independently from Sanofi Pasteur MSD. CC and NL are employed by Sanofi Pasteur MSD. AR, FB, GF and SV have no competing interests. PGR received travel reimbursement to present the results of this study in two conferences. AF received travel grants for conference attendance.

## Authors' contributions

PGR designed the study protocol, conducted the overall epidemiological analysis and wrote the study report. FP conducted the economic analysis and contributed to the report. AR collected the unit costs and contributed to the economic analysis. CC and NL contributed to the epidemiological and economic analyses and the study report. GF and SV conducted the literature reviews and helped collect the epidemiological data. AF contributed to the study design. All authors have approved the final version of the manuscript.

## Pre-publication history

The pre-publication history for this paper can be accessed here:


